# Spectral imaging and nucleic acid mimics fluorescence *in situ* hybridization (SI-NAM-FISH) for multiplex detection of clinical pathogens

**DOI:** 10.3389/fmicb.2022.976639

**Published:** 2022-09-29

**Authors:** Andreia S. Azevedo, Ricardo M. Fernandes, Ana R. Faria, Oscar F. Silvestre, Jana B. Nieder, Chenguang Lou, Jesper Wengel, Carina Almeida, Nuno F. Azevedo

**Affiliations:** ^1^LEPABE - Laboratory for Process Engineering, Environment, Biotechnology and Energy, Faculty of Engineering, University of Porto, Porto, Portugal; ^2^ALiCE - Associate Laboratory in Chemical Engineering, Faculty of Engineering, University of Porto, Porto, Portugal; ^3^i3S-Instituto de Investigação e Inovação em Saúde, Universidade do Porto, Porto, Portugal; ^4^IPATIMUP-Instituto de Patologia e Imunologia Molecular, Universidade do Porto, Porto, Portugal; ^5^INIAV, IP-National Institute for Agrarian and Veterinary Research, Vila Do Conde, Portugal; ^6^INL International Iberian Nanotechnology Laboratory, Av Mestre José Veiga s/n, Braga, Portugal; ^7^Biomolecular Nanoscale Engineering Center, Department of Physics, Chemistry and Pharmacy, University of Southern Denmark, Campusvej 55, Odense, Denmark

**Keywords:** nucleic acid mimics, fluorescence *in situ* hybridization, clinical pathogens, bacteria, multiplex detection, epifluorescence microscope

## Abstract

The application of nucleic acid mimics (NAMs), such as locked nucleic acid (LNA) and 2′-O-methyl-RNA (2’OMe), has improved the performance of fluorescence *in situ* hybridization (FISH) methods for the detection/location of clinical pathogens since they provide design versatility and thermodynamic control. However, an important limitation of FISH techniques is the low number of distinguishable targets. The use of filters in fluorescence image acquisition limits the number of fluorochromes that can be simultaneously differentiated. Recent advances in fluorescence spectral image acquisition have allowed the unambiguous identification of several microorganisms in a single sample. In this work, we aimed to combine NAM-FISH and spectral image analysis to develop and validate a new FISH variant, the spectral imaging-NAM-FISH (SI-NAM-FISH), that allows a multiplexed, robust and rapid detection of clinical pathogens. In the first stage, to implement/validate the method, we have selected seven fluorochromes with distinct spectral properties and seven bacterial species (*Pseudomonas aeruginosa*, *Citrobacter freundii*, *Staphylococcus aureus*, *Enterococcus faecalis*, *Klebsiella pneumoniae*, *Escherichia coli*, and *Acinetobacter calcoaceticus*). As a strong variation in fluorescence intensities is found between species and between fluorochromes, seven versions of a EUB LNA/2’OMe probe, each conjugated to one of seven fluorochromes, were used to rank species/fluorochromes by FISH and then optimize species/fluorochrome pairing. Then, final validation tests were performed using mixed populations to evaluate the potential of the technique for separating/quantifying the different targets. Overall, validation tests with different proportions of bacteria labeled with the respective fluorochrome have shown the ability of the method to correctly distinguish the species.

## Introduction

Fluorescence *in situ* hybridization (FISH) is a widely used molecular biology technique that is typically based on the hybridization properties of ribosomal RNA (rRNA) with a fluorescently label probe (usually composed of DNA) specifically designed for this purpose. The fluorescent signal emitted by the fluorochrome-probe complex allows for the hybridized probe to be detected since the target rRNA exists in sufficient copy numbers within the cell. This technique has been applied on a broad range of samples (e.g., biofilms, environmental, and medical samples) to quantify, visualize, and identify diverse microorganisms (Cerqueira et al., 2011; Azevedo et al., 2015; Rocha et al., 2019).

Despite the success of this tool in detecting microorganisms in a variety of samples, a significant drawback of FISH has been its low multiplex capability, as the number of distinguishable targets is usually limited to two or three targets (Valm et al., 2011, 2012). This limitation is related to the number of fluorochromes that can be discriminated using the standard filter setups of fluorescence microscopes. To solve this limitation, a FISH variation called combinatorial labeling and spectral imaging-FISH (CLASI-FISH) was developed. In the first version of this approach, 28 color combinations, each one for a specific organism, were generated using eight fluorochromes (Valm et al., 2011). This combinatorial labeling strategy, combined with spectral image acquisition and analysis, allowed to expand the number of distinguished targets in a unique image (Valm et al., 2011, 2012). Recently, the high-phylogenetic-resolution microbiome mapping by FISH (HiPR-FISH), which combines binary coding of up to 10 fluorochromes, spectral imaging, and machine learning, was capable of identifying 1,023 isolates of *Escherichia coli* (Shi et al., 2020). While these FISH variants, using rRNA-targeted probes, give us important phylogenetic information in complex microbial consortia, they are not able to provide information about the expressed genes. Hence, alternative FISH variants, such as sequential fluorescence *in situ* hybridization (seqFISH; Lubeck et al., 2014), spectral barcoding (Deng et al., 2018), spatial coding (Lubeck and Cai, 2012), and Multiplexed error-robust fluorescence *in situ* hybridization (MERFISH; Chen et al., 2015) aim to identify, count, and locate numerous mRNAs in a single cell.

Despite their great potential, all these FISH approaches have limitations related to the DNA and RNA probes, such as variable affinity of the different probes for their target, low robustness, and long hybridization periods (Yilmaz and Noguera, 2004; Yilmaz et al., 2006). To address these issues, several molecules that mimic nucleic acids (NAMs), such as locked nucleic acid (LNA) and 2′-O-methyl-RNA (2’OMe), can be used. NAMs have more recently been used for FISH with very promising results (Azevedo et al., 2015, 2019). They can display higher thermodynamic stability when hybridized with complementary DNA/RNA, which implies that shorter sequences can be used, enhancing the specificity of the hybridization (Azevedo et al., 2015). In addition, mixing of LNA nucleotides with DNA or other mimics greatly simplifies the design of multiplex approaches (detection of multiple targets simultaneously) by allowing a more complete control of the thermodynamics parameters (e.g., melting temperature (T_m_)-values and overall free energy change (ΔG_overall_)-values) of the different probes. In our previous studies using LNA/2’OMe-FISH, we demonstrated that it is possible to design probes for the detection of pathogenic bacteria at specific temperatures (e.g., human body temperature; Fontenete et al., 2013, 2016b), proving that probe affinity can be fine-tuned. In fact, the robustness of multiplex FISH approaches might be constantly improved with the use of LNA/2’OMe probes or other NAMs that may appear in future.

Herein, we combine the spectral imaging and NAM probes to develop a novel FISH methodology, named spectral imaging-NAM-FISH (SI-NAM-FISH) which allows a multiplexed, robust, and rapid detection of microorganisms. To our knowledge, this is the first time that NAM-FISH and spectral imaging are combined. In the first stage, the strategy to optimize the spectral imaging entails the use of an EUB338 LNA/2’OMe probe attached to seven different fluorochromes on a set of clinically relevant microorganisms (*Escherichia coli*, *Pseudomonas aeruginosa*, *Citrobacter* sp., *Staphylococcus aureus*, *Enterococcus* sp., *Klebsiella pneumoniae*, and *Acinetobacter* sp.). Then, species-specific LNA/2’OMe probes with similar affinity were used to show the capability of the SI-NAM-FISH to discriminate different targets.

## Results and discussion

### Implementation of SI-NAM-FISH using an EUB338 LNA/2’OMe probe attached to seven different fluorochromes

**Figure 1:**
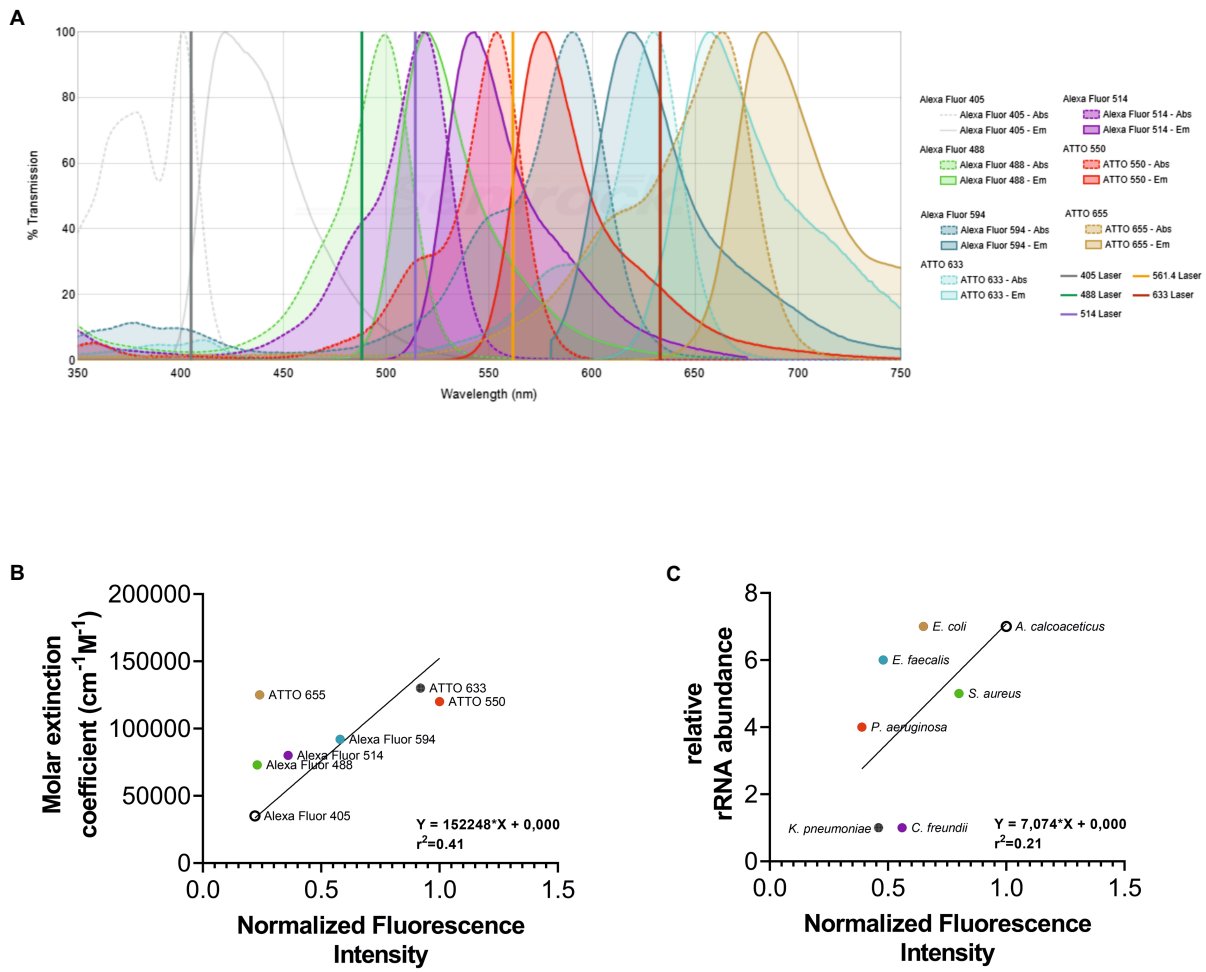
Representation of emission and excitation spectra of fluorochromes selected for this study. The fluorochromes selection was made according to their spectral properties and the filter setup of the fluorescence microscope to be used. As observed in **(A)** the emission spectra present a certain degree of the overlapping since the imaging strategy used in this methodology is based on the discrimination of the emission spectra of fluorochromes applying linear unmixing. To help on the selection of fluorochromes, the “SearchLight Spectra Viewer” from “Semrock” database (https://searchlight.semrock.com) was used. The normalized fluorescence intensity of each fluorochrome **(B)** and bacterium **(C)** was correlated with the molar extinction coefficient and the relative rRNA abundance, respectively. The fluorescence signal intensity of each image was determined using Fiji software. The molar extinction coefficient was obtained from data available by fluorochromes companies. The relative rRNA gene abundance of each bacterium was obtained on Greengenes database.

The first step of our work consisted of the selection of an appropriate set of fluorochromes. Fluorochromes were selected according to their spectral properties and the filter setup of the fluorescence microscope to be used. As the imaging strategy used in this methodology is based on the discrimination of the emission spectra of fluorochromes applying linear unmixing, it is still possible to distinguish different fluorochromes with a certain degree of the overlapping emission spectra (Figure 1A). In addition, according to the literature, the selected fluorochromes also presented high stability and brightness (Lavis and Raines, 2014). It is well known that fluorochromes with high quantum yields and molar extinction coefficients are brighter and more resistant to photobleaching (Lavis and Raines, 2014). However, their performance is influenced by the experimental settings and microscope setup used (e.g., optics, laser lines, type of detector). As such, fluorochromes were evaluated with a multiline confocal fluorescence using the LNA/2’OMe Eubacteria probe (EUB338) coupled to seven different fluorochromes. The probe was used to label separate aliquots of *E. coli* in FISH reactions. After the quantification of the fluorescence intensity signal from images for each fluorochrome, the results show that ATTO 550 and ATTO 633 exhibit the highest fluorescence intensity signal, and the ATTO 655, Alexa Fluor 488, and Alexa Fluor 405 present lower fluorescence intensity signal under the chosen excitation conditions for each fluorochrome (Figure 1B). These results are in general agreement with the molar extinction coefficient values for each fluorochrome at the respective excitation wavelength used (*r*^2^ = 0.41; Figure 1B). In addition, the relative brightness of fluorochromes (product of both molar extinction coefficient and quantum yield; Supplementary Figure S1) showed that the correlation coefficient is higher (*r*^2^ = 0.72).

On the other hand, a FISH signal also depends on the number of the fluorescently labeled probes able to hybridize with their target, which in this study is rRNA. It is well established that cells of different species have different numbers of rRNA molecules (Klappenbach et al., 2000). Hence, discrepant fluorescence intensity signals between different bacteria, with different characteristics and properties are expected to be observed. To evaluate the relative fluorescence intensity signal exhibited for each bacterium used in this study, an LNA/2’OMe probe EUB338 coupled to ATTO 550 was hybridized with each bacterium. Hybridization experiments were all performed at the exponential phase, as it is well stablished that ribosomes account for a significant volume fraction of exponentially growing cells. After the quantification of the fluorescence intensity, results show that *A. calcoaceticus* and *S. aureus* present the highest fluorescence intensity and *E. faecalis*, *K. pneumoniae*, and *P. aeruginosa* have the lowest fluorescence intensity signal (Figure 1C). The fluorescence intensity of each bacterium was correlated with the 16S rDNA gene copy numbers (that encodes for the 16 s rRNA sequences). However, the correlation coefficient is rather low (*r*^2^ = 0.21). The copy numbers of the 16S rDNA gene might not be related with the number of ribosomes in each bacterial cell; in fact, a single copy of 16S rDNA gene is clustered into a rRNA operon, with some bacterial species containing between 1 and 15 rRNA operons (Espejo and Plaza, 2018). In addition, the FISH signal intensity is also related with other factors, such as permeability of the cells and accessibility of the rRNA (Amann et al., 1995; Yilmaz and Noguera, 2004), which might also explain the low correlation coefficient.

**Figure 2:**
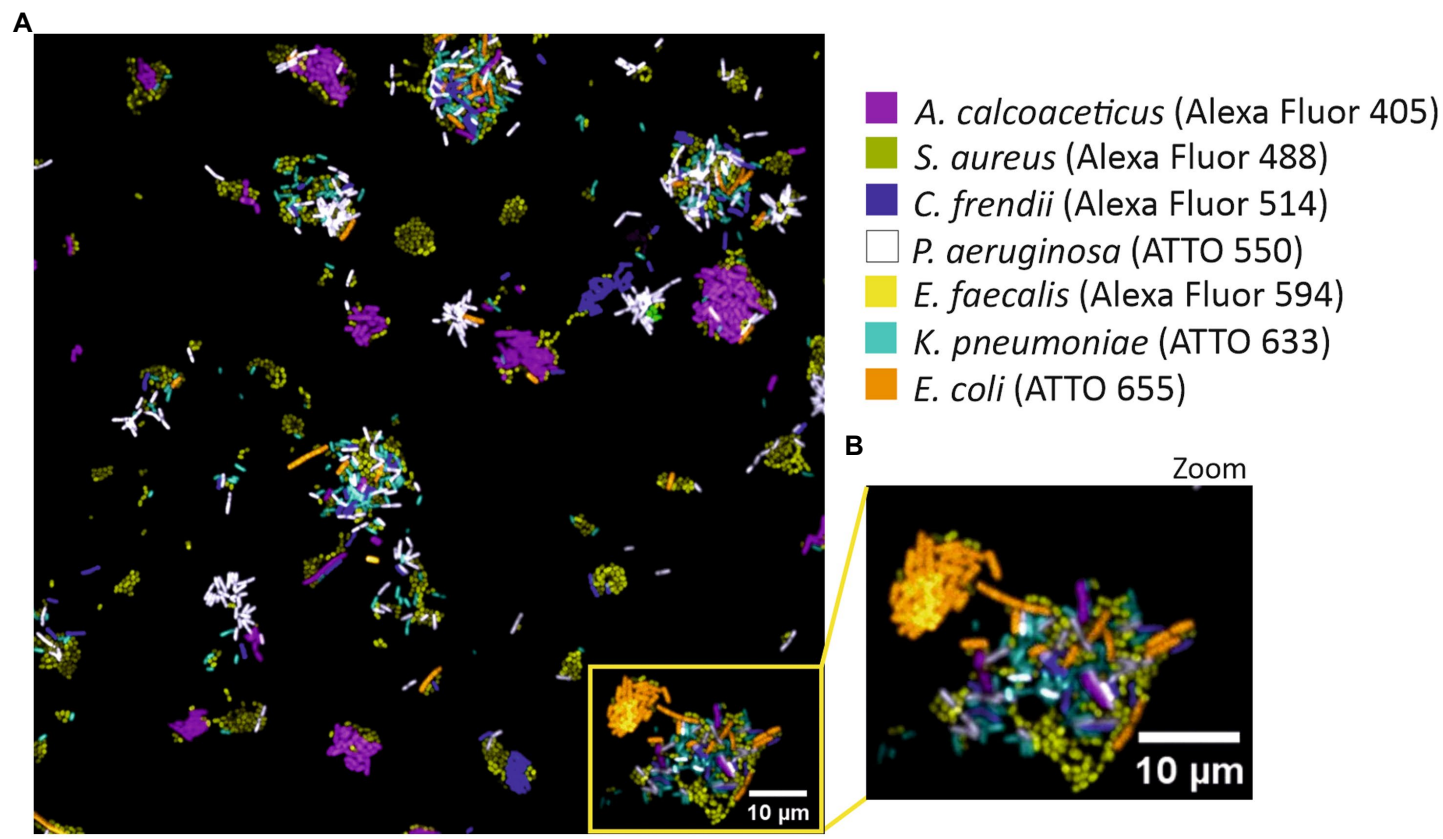
SI-NAM-FISH proof-of-principle with an EUB338 LNA/2’OMe probe attached to seven different fluorochromes. Unmixed image of a mixture of seven different bacteria labeled with an EUB338 LNA/2’OMe probe attached to ATTO 532 (magenta), Alexa Fluor 488 (green), Alexa Fluor 514 (blue), ATTO 550 (white), Alexa Fluor 594 (yellow), ATTO 633 (cyan) and ATTO 655 (orange). The image corresponds to a merge of all seven fluorochromes channels after linear unmixing **(A)**. Enlarged part of the image **(B)** as indicated by the frame in panel **(A)**. The bacteria, fluorochromes, and assigned false colors are indicated.

For the success of SI-NAM-FISH, the fluorescence intensity signals must be similar (Cohen et al., 2018). As such, fluorochromes with high initial brightness were used to detect bacteria targets with the lowest fluorescence (Supplementary Table S1). To confirm that the above-selected bacteria species/fluorochrome pairs could be distinguished in a spectrally acquired microscope image, each version of the LNA/2’OMe probe EUB338 was hybridized with each allocated bacterium individually (Supplementary Table S1). After FISH, the separately labeled bacterial samples were combined and imaged spectrally. Results show that all bacteria are clearly and specifically identified (Figure 2).

### LNA/2’OMe species-specific probes design and optimization

**Figure 3:**
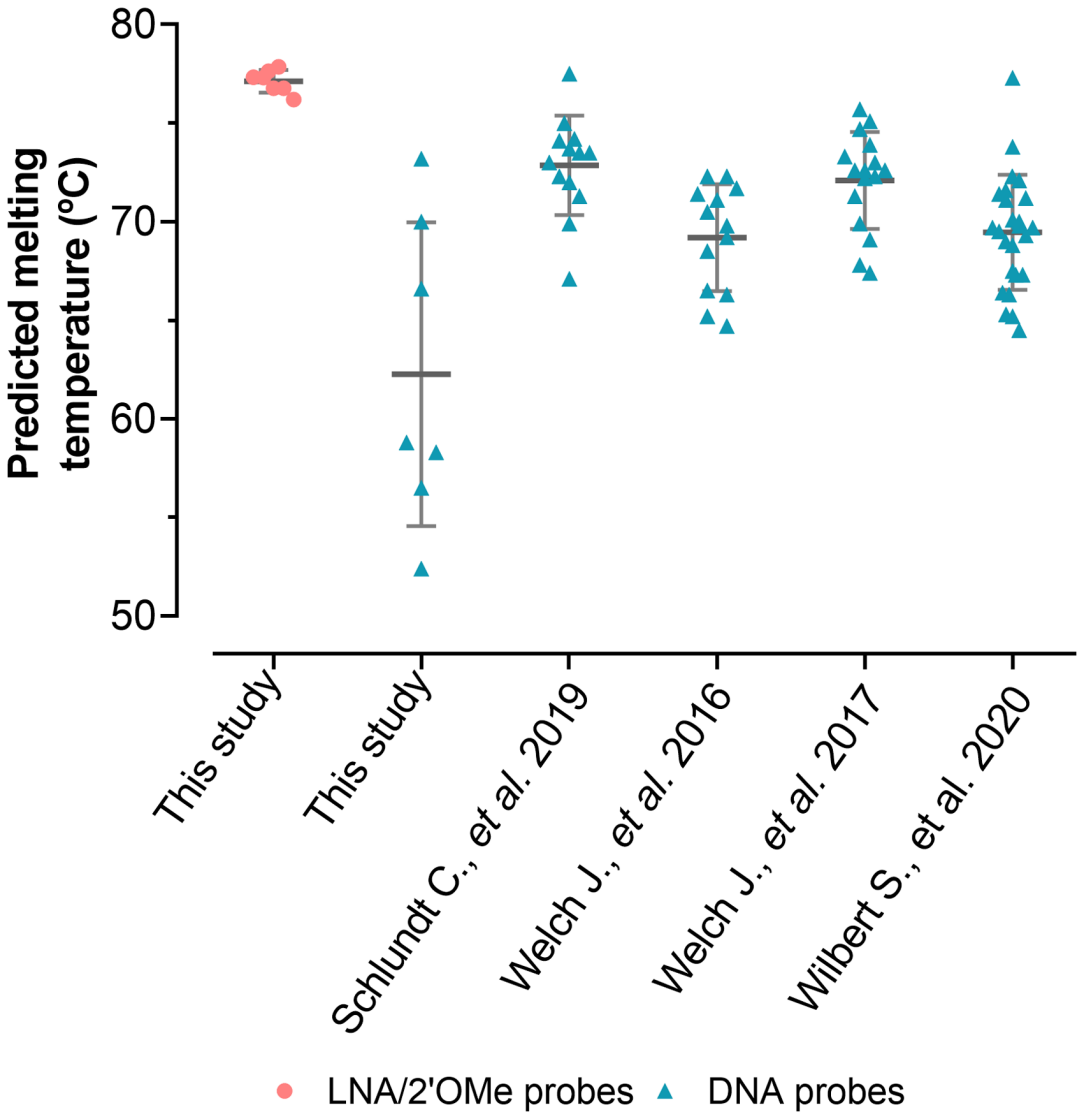
Comparison between predicted melting temperatures of the LNA probes used in this study and DNA probes already published (16–19). The DNA probes of this study have the exact same sequences of the LNA probes but were only used for the predicted melting temperature assessment. Predicted melting temperatures were calculated according to Fontenete et al. (2016a).

**Figure 4:**
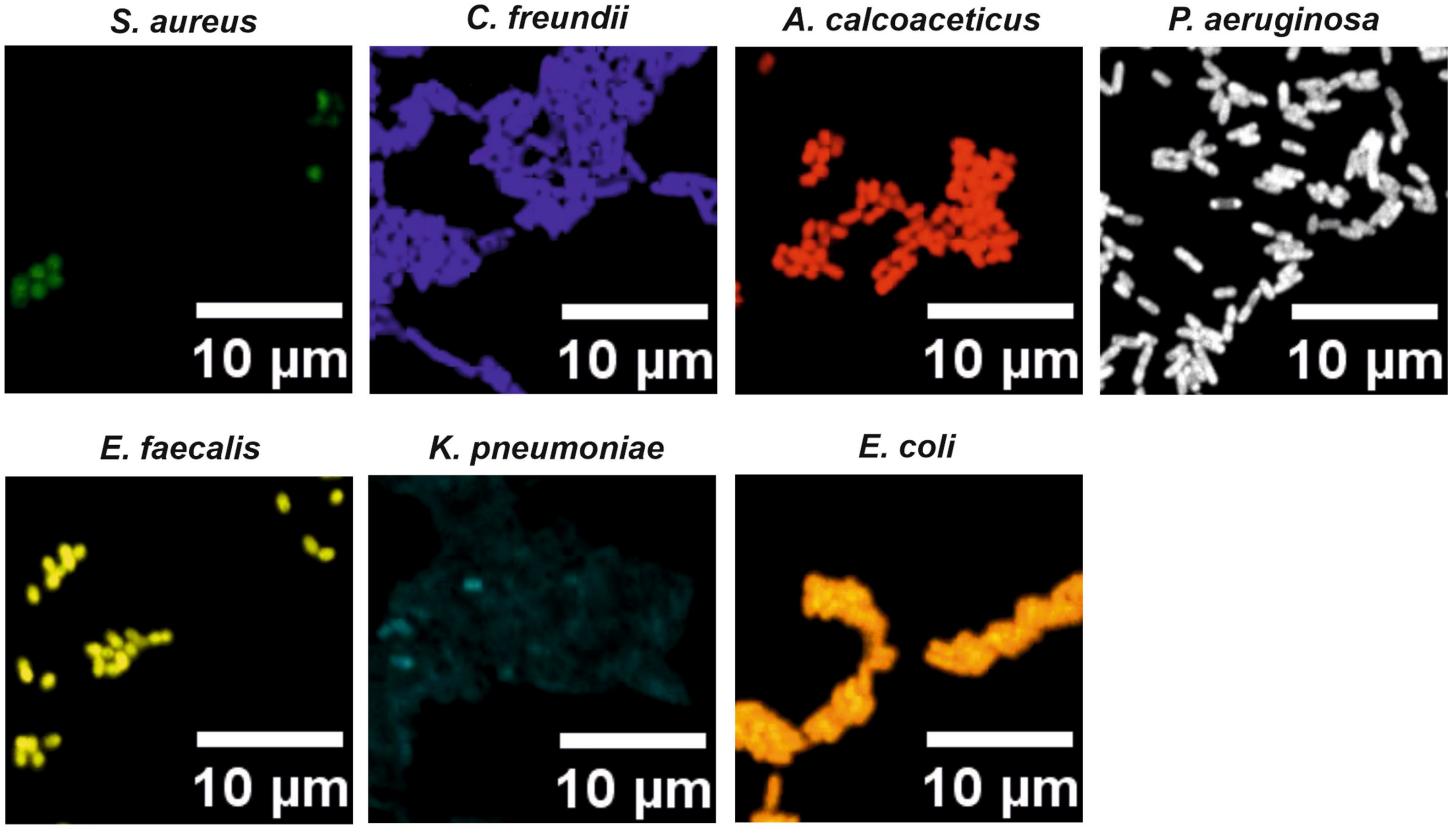
Hybridization results for the species-specific LNA/2’OMe probes with pure cultures smears. A range of hybridization temperatures, between 50 and 65°C, was tested to achieve the best FISH signals for a multiplex assay. The same hybridization conditions were used for all probes. The hybridization temperature selected for the multiplex experiments was 62°C.

For the specific LNA/2’OMe probes targeting the species and the genus of interest, reported sequences in the literature were re-evaluated, or new sequences were designed. All selected sequence probes presented high specificity (>99.99%) and high sensitivity (>90.00%; Supplementary Table S2). In addition, thermodynamic parameters were also considered, including the ΔG_overall_ and the T_m_. The T_m_ values should be similar in order to increase the chances of the probes to hybridize under similar conditions in a multiplex assay. The insertion of LNA and 2’OMe-RNA in the probe design allowed for a set of probes with very similar theoretical T_m_-values (76–77°C; Supplementary Table S2), indicating that these probes should hybridize under similar conditions. The predicted T_m_ values (Figure 3) also demonstrate that, for the equivalent DNA sequences, the T_m_ values are lower and much more diverse. The adjustment of the length of DNA probes is the only way to bring their thermodynamic parameters to similar values. This makes the design of multiplex approaches much more limited, and consequently, the signal intensity of the probes might be compromised. The species-specific LNA/2’OMe probes were then tested and optimized through well-established FISH procedures (Azevedo et al., 2019). The results show the effectiveness and specificity of the LNA/2’OMe species-specific probes at 62°C (Figure 4); in fact, the temperature range of 60°C-65°C also provided a high signal-to-noise ratio and could alternatively be used for multiplex approaches (data not shown).

### SI-NAM-FISH detects the seven bacterial species with high specificity and sensitivity

**Figure 5:**
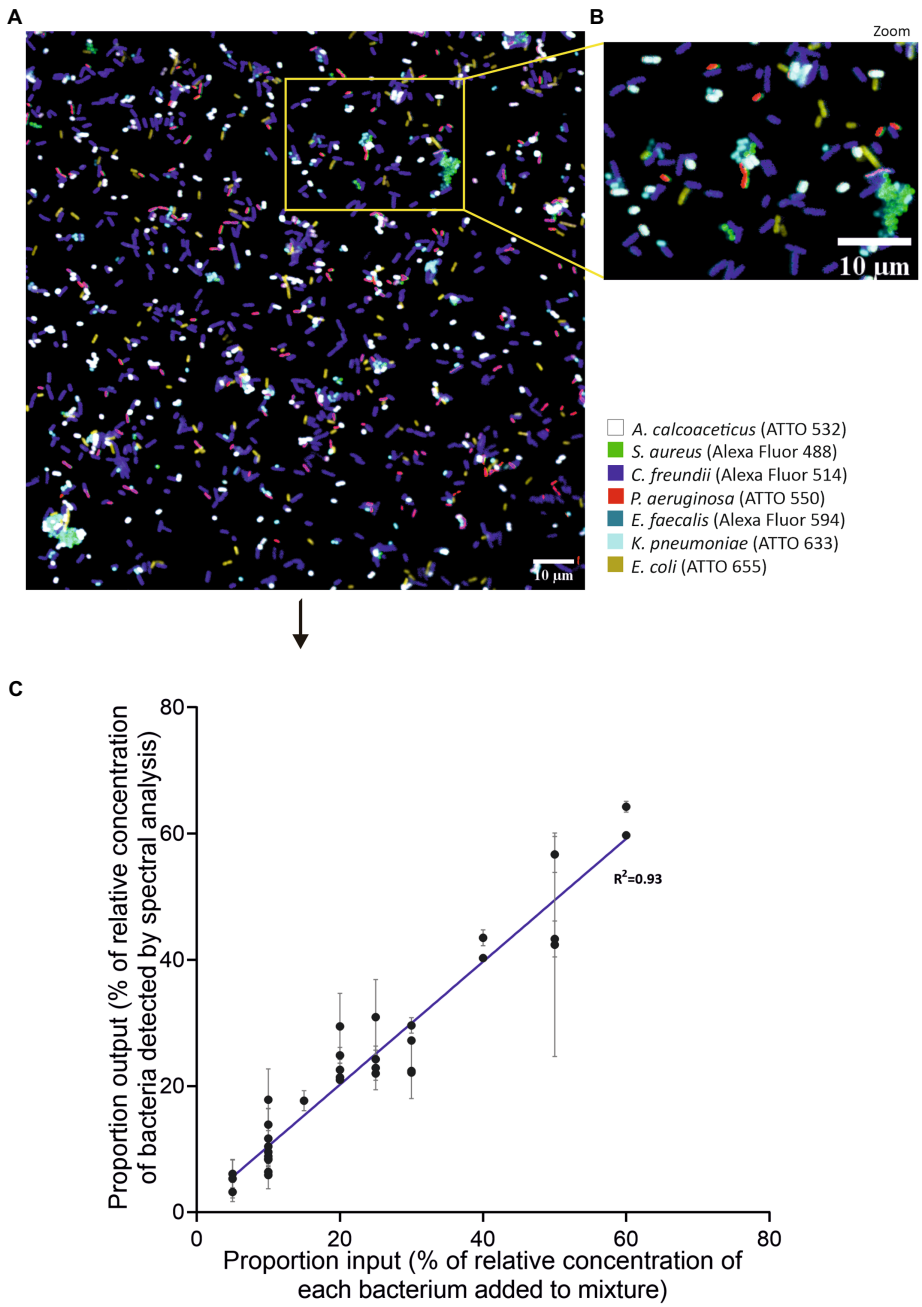
Validation of SI-NAM-FISH proof-of-principle using seven species-specific LNA/2’OMe probes. Unmixed image of a mixture of seven different bacteria labeled species-specific LNA/2’OMe probes attached to Alexa Fluor 488 (green, *S. aureus*), Alexa Fluor 514 (magenta, *C. freundii*), ATTO 532 (white, *A. calcoaceticus*), ATTO 550 (red, *P. aeruginosa*), Alexa Fluor 594 (blue, *E. faecalis*), ATTO 633 (turquoise, *K. pneumoniae*), and ATTO 655 (yellow, *E. coli*). The image corresponds to a merge of all seven fluorochromes channels after linear unmixing **(A)**. Enlarged part of the image **(B)** as indicated by the frame in panel **(A)**. The bacteria, fluorochromes, and assigned false colors are indicated. Correlation between the percentage of relative concentration of each bacterium added to the mixture and the percentage of relative concentration of bacteria detected by spectrally acquired microscope images after application of a linear unmixing algorithm **(C)**. Error bars represent standard deviation from two different fields of view of the same artificial mixture.

We next tested the SI-NAM-FISH as a methodology to identify and distinguish seven different species in an artificial mixture using species-specific LNA/2’OMe probes. At this stage, adjustments on confocal settings such as laser intensities and gain were carried out to reach a desired performance of the methodology (Supplementary Table S1). Figures 5A,B represent an unmixed image of a mixture of seven different bacteria labeled with the species-specific LNA/2’OMe probes. To assess the accuracy of the SI-NAM-FISH, the protocol was applied to different mixtures containing known concentrations of each bacterium (Supplementary Figure S2). Gratifyingly, a good correlation was obtained between the input values and the output values (Figure 5C). Together, these data confirm the accuracy of the SI-NAM-FISH to identify and correctly distinguish at least seven microorganisms in a complex population.

Overall, all these data highlight the added value of combining NAM-FISH with spectral imaging for multiplex approaches. While CLASI-FISH using combinatorial labeling can differentiate more than eight organisms, this technique suffers from limitations associated with the use of DNA/RNA probes [low affinity (Yilmaz and Noguera, 2004) and long hybridization steps (Valm et al., 2011; Schlundt et al., 2019)], which might compromise the procedure robustness and hinders broader implementation. In CLASI-FISH, using multiple probes to label each microorganism requires a similar performance of the probes. By using DNA or RNA probes, these requirements might not be met, decreasing the capabilities of detection and discrimination of microorganisms. The present study shows that using LNA/2’OMe probes and a simple approach that makes use of single labeling, instead of a binary labeling, we can accurately discriminate several bacterial species with different properties. In addition, using such NAMs, the hybridization time is shorter; in fact, the hybridization protocol here implemented is faster (90 min) than multiplex DNA/RNA-FISH protocols described in the literature (3–4 h; Valm et al., 2011; Schlundt et al., 2019). Despite the higher costs of NAMs comparing to DNA or RNA probes, they might enhance the performance of FISH methods, showing high affinity of the probe for its target and high robustness.

**Figure 6:**
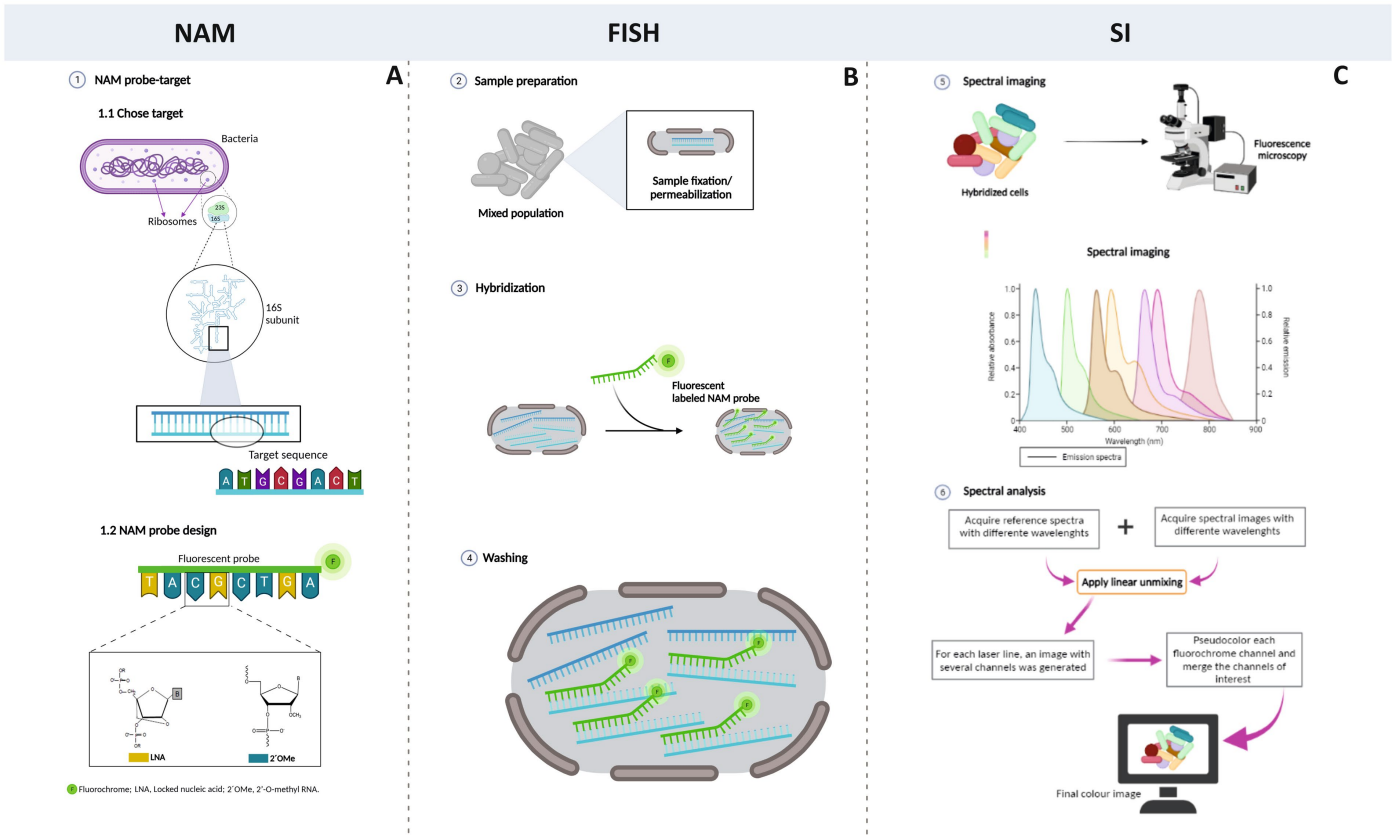
Schematic representation of SI-NAM-FISH. The first step involves the selection of the target and the design of the NAM probe **(A)**; then, the FISH procedure **(B)** is optimized. For that, the sample is fixed and permeabilized to preserve the cell structure and to ensure the probe entry into the cells. Then, a specific fluorescent NAM probe binds to rRNA that exist in high copy number within the cell; afterward, the washing step allows to remove the unbound NAM probe or the excess of the probe. The panel B illustrates the hybridization process for one probe; however, in the present work, a multiplex approach is applied. As different fluorochromes present a certain degree of the overlapping emission spectra, the spectral imaging strategy is used **(C)**. The sample is illuminated sequentially with each wavelength excitation (in order of descending wavelength of the excitation laser light). Images are processed by applying a linear unmixing algorithm with reference spectra for each excitation wavelength. This process compiles each single-labeled bacteria image in a final common image where different species are discriminated.

This is the first time that the fusion between NAM-FISH and spectral imaging (Figure 6) is implemented, making this a very innovative and challenging work. This new FISH variant is highly suitable for a multiplex, robust, and rapid detection of relevant clinical pathogens and it can also be of great use on the study of complex microbial communities, such as biofilms, to study microbial-microbial and microbial-host interactions at the diversity level and spatial level.

## Materials and methods

### Microbial strains and culture maintenance

The bacterial strains selected for this study were *Escherichia coli* CECT 515, *Pseudomonas aeruginosa* PAO1, *Citrobacter freundii* SGSC 5345, *Staphylococcus aureus* ATCC 25523, *Enterococcus faecalis* CECT 184, *Klebsiella pneumoniae* ATCC 11269, and *Acinetobacter calcoaceticus* Aba 1. All bacterial strains were grown on tryptic soy agar (TSA) [3% (wt/vol) tryptic soy broth and 1.5% (wt/vol) agar; Liofilchem, Italy] at 37°C and streaked onto fresh plates every 24 h.

### Implementation of SI-NAM-FISH using a universal EUB 338 LNA/2’OMe probe

In the first stage, a universal EUB338 LNA/2’OMe probe (5’-UgCCtCCcGUaGGa-3′; LNA nucleotide monomers are represented in lowercase (the base in LNA-C is 5-methylcytosine) and 2’-OMe-RNA monomers are represented in capital letters) with a phosphorothioate (PS) backbone, and an amino-C6 at the 5′-end needed for the amide labeling with the respective fluorochromes was used in order to optimize and validate the SI-NAM-FISH. The probe sequence design was based on Amann et al. (1990). EUB338 LNA/2’OMe attached to ATTO 550, ATTO 633, and ATTO 655, respectively, were synthesized at the Biomolecular Nanoscale Engineering Center (SDU, Denmark); and EUB338 LNA/2’OMe attached to Alexa Fluor 405, Alexa Fluor 488, Alexa Fluor 514 and Alexa Fluor 594, respectively, were purchased from Eurogentec (Belgium).

#### Matching of the fluorochromes with bacteria for balanced fluorescence signal

For a successful SI-NAM-FISH, a balanced bacteria/fluorochromes pairs should be established to enable clear and unambiguous separation of the fluorochromes in a multiplex assay. Hence, seven versions of the EUB338 LNA/2’OMe probe attached to different fluorochromes (Alexa Fluor 405, Alexa Fluor 488, Alexa Fluor 514, ATTO 550, Alexa Fluor 594, ATTO 633, and ATTO 655) were used to rank the fluorochromes. For that, these EUB338 LNA/2’OMe probe versions were used to label separate aliquots of *E. coli* cells in FISH reactions. On the other hand, to rank the bacteria, a EUB338 LNA/2’OMe probe coupled with ATTO 550 was used to label separate aliquots of *E. coli* CECT 515, *P. aeruginosa* PAO1, *C. freundii* SGSC 5345, *S. aureus* ATCC 25523, *E. faecalis* CECT 184, *K. pneumoniae* ATCC 11269, and *A. calcoaceticus* Aba 1 in FISH reactions. The FISH protocol was performed according to Azevedo et al. (2019). Briefly, cellular suspensions from each species at the exponential phase were centrifuged (10.000 x *g*, 5 min) and fixed in 400 μl of 4% (v/v) paraformaldehyde (Sigma, United States) for 1 h at room temperature. Then, fixed cells were resuspended in 500 μl of 50% (vol/vol) ethanol and incubated at −20°C for at least 30 min. After the fixation and permeabilization steps, 100 μl of fixed cells was mixed in 100 μl of hybridization solution [2 M Urea (National Diagnostics, United States), 4 M NaCl (Fisher Scientific, Belgium), and 50 mM Tris–HCl (Sigma, United States), pH 7.5] with 200 nM of the probe and incubated at 60°C for 60 min (Azevedo et al., 2019). Afterward, samples were centrifuged (10.000 x *g*, 5 min), resuspended in 500 μl of washing solution containing 5 mM Tris-base (pH 10; Sigma, United States), 15 mM NaCl (Fisher Scientific, Belgium), and 0.1% (v/v) Triton X-100 (Sigma, United States) and incubated for 30 min at 60°C. Subsequently, the suspension was pelleted by centrifugation and resuspended in sterile water. Additionally, for each experiment, a negative control was performed simultaneously, without the addition of the probe. After FISH, 10 μl of each cell suspension were put on a microscope slide coated with poly-L-lysine (Sigma, United States) and allowed to air dry, protected from the light. Finally, the samples were mounted with one drop of Fluoromount Aqueous Mounting Medium (Sigma, United States). The samples were stored at 4°C in the dark for 24 h before microscopy analysis. Three independent experiments were performed.

Samples were imaged with a Zeiss LSM 780 laser scanning confocal microscope equipped with five laser lines (405, 488, 514, 561, and 633 nm). The lambda mode of acquisition and the Plan-Apochromat 63x/1.4 Oil DIC M27 (FWD = 0.19 mm; Zeiss, Germany) immersion oil objective was used to acquire the images. Laser power was optimized for each fluorochrome assigned to each target to maximize signals without saturation. The fluorescence intensity of each image was quantified using the Fiji software (Wayne Rasband National Institutes of Health). This quantification allowed us to determine the average fluorescence intensity of each image.

#### Optimization of SI-NAM-FISH

To implement the SI-NAM-FISH, a preliminary experiment was performed in order to evaluate the ability of the method to correctly distinguish the different fluorochromes with highly overlapping emission spectra. Here, separate aliquots of each bacterium were hybridized with a different version of the EUB338 LNA/2’OMe probe, using the matches species/fluorochromes defined above. The FISH protocol was performed as already described. Then, a mixture was performed and was spotted onto a slide coated with poly-L-lysine and the samples. Finally, the sample was mounted with one drop of Fluoromount Aqueous Mounting Medium for imaging. Single samples, each labeled with a different fluorophore version of the EUB-338 probe, were used to create the spectral reference standards.

#### Spectral image acquisition and image analysis

Samples were imaged with a Zeiss LSM 780 laser scanning confocal microscope equipped with a 32-channel multianode spectral detector and five laser lines (405, 488, 514, 561, and 633 nm). Sequential spectral images were acquired with the Plan-Apochromat 63x/1.4 Oil DIC M27 (FWD = 0.19 mm) lambda mode of acquisition, with 8.8 nm channel widths, and from 411 to 692 nm in order of descending wavelength of the excitation laser light (633, 561, 514, 488, and 405 nm). For each laser line, the laser intensity and gain were adjusted to get the best possible signal-to-noise ratio without saturation. At least two fields of view per sample were imaged to acquire the spectral images for each laser line. In addition, for each fluorochrome, the reference spectra from single-labeled samples were acquired under the same conditions and with the same settings (same objective, multiline beam dichroic, detector bandwidth settings, gain and power per laser line) used for acquiring spectral images. Then, using the Zeiss ZEN software, a region of interest (ROI) in an area without cells was defined as the background region for each image. The Autofind/ACE function automatically generated the reference spectrum and, subsequently, each spectral image obtained for each laser line was unmixed separately with the respective reference spectrum of fluorochromes that are best excited by each laser line. More specifically, for each excitation image, the following reference spectra were included: 633 nm excitation image: Alexa Fluor 594, ATTO 633, and ATTO 655; 561 nm excitation image: Alexa Fluor 514, ATTO 550, Alexa Fluor 594, ATTO 633, and ATTO 655; 514 nm excitation image: Alexa Fluor 488, Alexa Fluor 514, ATTO 550, and Alexa Fluor 594; 488 nm excitation image: Alexa Fluor 488, Alexa Fluor 514, and ATTO 550; and 405 nm excitation image: Alexa Fluor 405. The results from off-peak fluorochromes were discarded as preliminary analysis indicated that the final images were not affected (these discarded channels presented a lower signal-to-noise compared with the channels from the more favorable excitation wavelength). For each laser line, an image with several channels was generated, in which each channel corresponds to a different fluorochrome. Finally, the channels of interest were merged to generate a final color image.

### LNA/2’OMe probes development

Species-specific probes will target conserved regions in the 16S or 23S rRNA. Before proceeding with probe design, the “probeBase”^1^ was used to identify probes that have already been reported in the literature. For bacteria with reported probes, sequences were re-evaluated in terms of theoretical specificity and sensibility. For the design of new probes, an approach using available alignment programs coupled with the 16S/23S rRNA databases was used according to the methodology described before (Teixeira et al., 2021). In short, 16S and 23S rRNA gene sequences available at ARBSilva^2^ were selected. For each organism, this selection contained at least 10 sequences of the target species and at least seven sequences of related species belonging to the target family. Then, Geneious Prime was used to identify conserved regions among the target species sequences.

The criteria for selecting the final probe sequence included the length, percentage of GC, number of LNA/2’OMe residues, and lack of self-complementary and secondary structures. In addition, thermodynamic parameters of the probes assessed, such as the Tm and ΔG_overall_, were determined according to Fontenete et al. (2016a).

Then, the theoretical sensitivity and specificity of the probes were evaluated according to Teixeira et al. (2021). Briefly, to evaluate the theoretical sensitivity and specificity of the probes, the TestProbe program was used.^3^ Specificity was calculated as nTs/(TnT) × 100, in which nTs represent the total number of non-target sequences that did not react with the probe and TnT is the total non-target sequences present in the database. Sensitivity was calculated as Ts/(TTs) × 100, where Ts is the number of target sequences detected by the probe and TTs is the total number of target sequences present in the database. In addition, to prevent self-complementarity of the sequences, the probes were evaluated on the online tool DINAMelt Web Server and the Quickfold tool^4^ to predict secondary structures. Lastly, the designed LNA/2’OMe probes coupled to different fluorochromes were synthesized at Eurogentec (Belgium).

### Detection of clinical pathogens using the species-specific LNA/2’OMe probe and spectral imaging analysis

First, the species-specific LNA/2’OMe probes were optimized on pure samples according to the FISH procedure described above. Then, different hybridization temperatures, between 50°C and 65°C, were tested to achieve the best FISH signals for a multiplex assay. After the FISH protocol, the samples were analyzed by a Zeiss LSM 780 laser scanning confocal microscope. For each probe, the adequate laser line was used, and the power of the laser and the gain were optimized for the highest possible signal-to-noise ratio. The fluorescence intensity of each species-specific LNA/2’OMe probe at different temperatures was quantified using the Fiji software as previously used.

After the selection of the best hybridization conditions, the discrimination power of the methodology was evaluated. For that, a correlation between known input (relative concentration of each bacterium added to the initial mixture) and output (relative concentration of each bacterium detected by spectral imaging after applying of an unmixing algorithm) was performed. Then, different mixtures with different known concentrations of each label-bacterium were hybridized with a mix of species-specific LNA/2’OMe probes, using the matches species/fluorochromes defined above (Supplementary Table S3). Next, the FISH protocol was performed as already described (section “Matching of the fluorochromes with bacteria for balanced fluorescence signal”). Afterward, the mixtures were spotted onto a slide coated with poly-L-lysine, and the samples were mounted with one drop of Fluoromount Aqueous Mounting Medium for imaging. Pure samples, each labeled with a species-specific LNA/2’OMe probe, were used to create the spectral reference standards.

The spectral imaging acquisition was performed as described above (section “Implementation of SI-NAM-FISH”). At this stage, adjustments regarding the settings of confocal (laser intensity and gain) are expected to be necessary. As described in section “Spectral image acquisition and image analysis,” spectrally acquired images were subjected to linear unmixing available on microscope processing software (Zeiss ZEN software). This was an automated analysis, in which the spectrally recorded images were unmixed separately with the respective reference spectrum of fluorochromes to assign to each pixel in the image its fluorochrome composition. This process involves the estimation of the proportion of different fluorochromes in each pixel of the images. As described above, to generate reference spectra, a region of interest (ROI) was specified, and the software displayed the mean intensity of all pixels within the ROI versus the wavelength. Then, to determine the spectral content of each pixel in an image, the software match/compare the summed spectrum from that pixel against a library of reference spectra according to the best-fit parameters mandated by the software. After the images have been unmixed, the software averaged the intensity values for every pixel that make up each cell. In this case, an average value for all fluorochromes used in the experiment was obtained and the channels with the highest average fluorochrome abundance at each cell allowed identify that object’s fluorochrome composition. The result was multi-fluorescence images with clear separated image channels representing one fluorochrome each. The images of interest were merged to generate a final color image. For each species/fluorochrome, a pseudocolor was attributed and counted. The correlation value, *r*, was calculated as the Pearson’s correlation coefficient between the mean values of relative concentration of each bacterium in mixed samples and mean values of relative concentration obtained in spectral images. Finally, it is expected to obtain a good correlation between the relative concentration of each bacterium in mixed samples and the relative concentration obtained in spectral images.

## Data availability statement

The datasets presented in this study can be found in online repositories. The names of the repository/repositories and accession number(s) can be found in the article/Supplementary material.

## Author contributions

AA and CA contributed to the conception and design of the study. AA, RF, and AF performed the experiments and analyzed the data. AA wrote the manuscript. JN, CA, and NA provided assistance and guidance throughout the research. JW and CL assisted in the synthesis of LNA probes. ÓS, JN, JW, CA, and NA revised the manuscript. All authors contributed to the article and approved the submitted version.

## Funding

This work was financially supported by: LA/P/0045/2020 (ALiCE), UIDB/00511/2020, and UIDP/00511/2020 (LEPABE), funded by national funds through FCT/MCTES (PIDDAC); Project POCI-01-0145-FEDER-030431 (CLASInVivo), funded by FEDER funds through COMPETE2020 – Programa Operacional Competitividade e Internacionalização (POCI) and by national funds (PIDDAC) through FCT/MCTES and NORTE-01-0145-FEDER-000019 (Nanotechnology based functional solutions) and by national funds (PIDDAC) through FCT/MCTES. We thank the Nanophotonics and Bioimaging Facility (NBI) at the International Iberian Nanotechnology Laboratory (INL) for support. OS received a fellowship *via* the project NanoTRAINForGrowth, FP7-PEOPLE-COFUND-FP program (grant no: 600375).

## Conflict of interest

The authors declare that the research was conducted in the absence of any commercial or financial relationships that could be construed as a potential conflict of interest.

## Publisher’s note

All claims expressed in this article are solely those of the authors and do not necessarily represent those of their affiliated organizations, or those of the publisher, the editors and the reviewers. Any product that may be evaluated in this article, or claim that may be made by its manufacturer, is not guaranteed or endorsed by the publisher.

## Footnotes

^1^ http://www.probebase.net

^2^ https://www.arb-silva.de

^3^ https://www.arb-silva.de/search/testprobe/

^4^ http://mfold.rna.albany.edu/?q=DINAMelt/Quickfold
