# Supplementary material for: Spectral imaging and nucleic acid mimics fluorescence in situ hybridization (SI-NAM-FISH) for multiplex detection of clinical pathogens
Source: Front Microbiol. 2022 Sep 29;13:976639. doi: 10.3389/fmicb.2022.976639 (PMC9557775; doi:10.3389/fmicb.2022.976639)
Supplement: Supplementary file 1 [file Data_Sheet_1.docx]

Supplementary Material

**Table S1.** The balanced species/fluorochromes pairs and settings used for imaging on confocal microscope.

| **Fluorochrome labelled** | **Target/Probe** | **Confocal settings** | | |
| --- | --- | --- | --- | --- |
|  |  | **Laser** | **Laser intensity** (**%)** | **Gain** |
| ATTO 550 | *P. aeruginosa* | 561 | 2 | 560 |
| ATTO 633 | *K. pneumoniae* | 633 | 15 |  |
| Alexa Fluor 594 | *Enterococcus sp.* | 561 | 2 |  |
| Alexa Fluor 514 | *Citrobacter sp.* | 514 | 5 |  |
| ATTO 655 | *E. coli* | 633 | 15 |  |
| Alexa Fluor 488 | *S. aureus* | 488 | 5 |  |
| ATTO 532 | *Acinetobacter sp.* | 514 | 5 |  |

**Table S2.** Species-specific LNA/2’OMe probes sequences used in this study and their properties.

| **Target organism** | **rRNA**  **subunit** | **LNA/2’OMe probe sequence (5´-3´)** | **% GC** | **Melting temperature**  **°C** | **ΔG_overall_**  **Kcal/mol** | **Specificity**  **%** | **Sensibility**  **%** | **Reference** |  |
| --- | --- | --- | --- | --- | --- | --- | --- | --- | --- |
| *E. coli* | 16S | AcGTcAAtGAgCAaAGg | 47.06 | 76.77 | -29.35 | 99.92 | 92.09 | Juhna et al. 2007 | |
| *P. aeruginosa* | 23S | cTTgAAa**c**cCCgGAt^1^ | 53.33 | 77.32 | -27.63 | 99.96 | 100.00 | This study | |
| *Citrobacter sp.* | 16S | cCAgTTt**c**gGAtGC^2^ | 57.14 | 77.85 | -27.88 | 99.92 | 68.99 | This study | |
| *S. aureus* | 16S | AgCAaGCtTCtCGtCC | 56.25 | 77.63 | -32.86 | 99.99 | 93.68 | Kempf et al. 2000 | |
| *Enterococcus sp.* | 23S | gTTcTCtGCgTCtACcTC | 55.56 | 77.31 | -38.40 | 99.86 | 94.29 | Wellinghausen et al. 2007 | |
| *K. pneumoniae* | 23S | cTAcACa**c**cAGcGT^3^ | 57.14 | 76.19 | -30.90 | 99.89 | 99.05 | Kempf et al. 2000 | |
| *Acinetobacter sp.* | 16S | TcCTcTCcCAtACtCT | 50.00 | 76.77 | -32.04 | 99.99 | 97.09 | Wagner et al. 1994 | |

LNA nucleotide monomers are represented at lowercase; 2’-OMe-RNA monomers are represented at capital letters.

^1^One mismatch for other *Pseudomonas;* ^2^One mismatch for *Pseudomonas* sp.; ^3^One mismatch for *Enterobacter* sp..


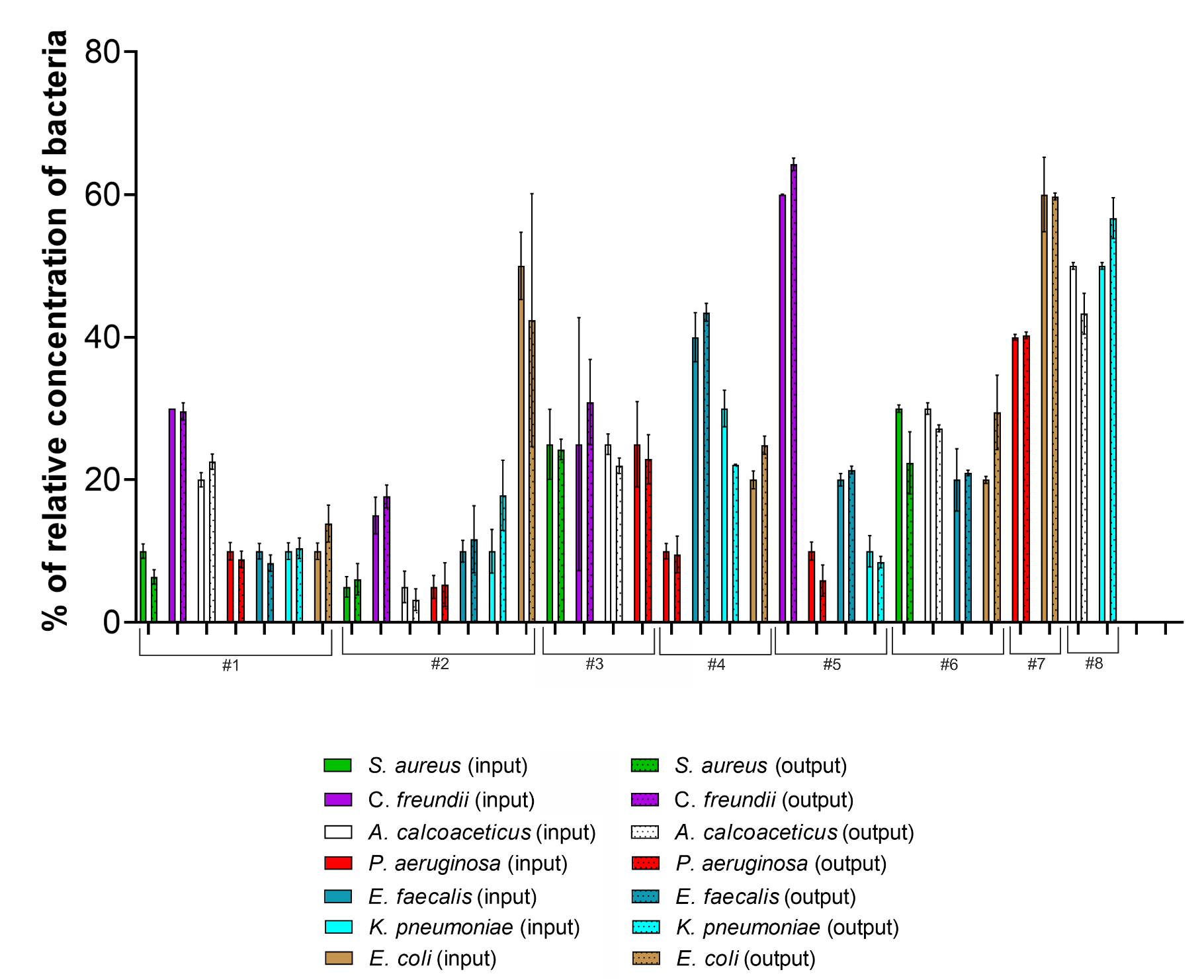


**Figure S2.** Quantification of % of relative concentration of bacteria (output) into mixtures after spectral image acquisition. The bars without pattern bars represent input into the mixture (% of relative concentration of each bacterium added to the mixture), and bars with pattern represent output into the mixture. Error bars in the output measurements represent SD in the percent of each species counted in at least two fields of view per sample of SI-NAM-FISH-labeled bacteria.

| **Mix** | **Bacteria** | **Proportion input (%)** |
| --- | --- | --- |
| #1 | *S. aureus* | 10 |
|  | *C. freundii* | 30 |
|  | *A. calcoaceticus* | 20 |
|  | *P. aeruginosa* | 10 |
|  | *E. faecalis* | 10 |
|  | *K. pneumoniae* | 10 |
|  | *E. coli* | 10 |
| #2 | *S. aureus* | 5 |
|  | *C. freundii* | 15 |
|  | *A. calcoaceticus* | 5 |
|  | *P. aeruginosa* | 5 |
|  | *E. faecalis* | 10 |
|  | *K. pneumoniae* | 10 |
|  | *E. coli* | 50 |
| #3 | *S. aureus* | 25 |
|  | *C. freundii* | 25 |
|  | *A. calcoaceticus* | 25 |
|  | *P. aeruginosa* | 25 |
| #4 | *P. aeruginosa* | 10 |
|  | *E. faecalis* | 40 |
|  | *K. pneumoniae* | 30 |
|  | *E. coli* | 20 |
| #5 | *C. freundii* | 60 |
|  | *P. aeruginosa* | 10 |
|  | *E. faecalis* | 20 |
|  | *K. pneumoniae* | 10 |
| #6 | *S. aureus* | 30 |
|  | *A. calcoaceticus* | 30 |
|  | *E. faecalis* | 20 |
|  | *E. coli* | 20 |
| #7 | *P. aeruginosa* | 40 |
|  | *E. coli* | 60 |
| #8 | *A. calcoaceticus* | 50 |
|  | *K. pneumoniae* | 50 |

**Table S3.** The mixtures of bacterial species and respective % of relative concentration of each bacterium added to the mixture used to assess the accuracy of the SI-NAM-FISH.


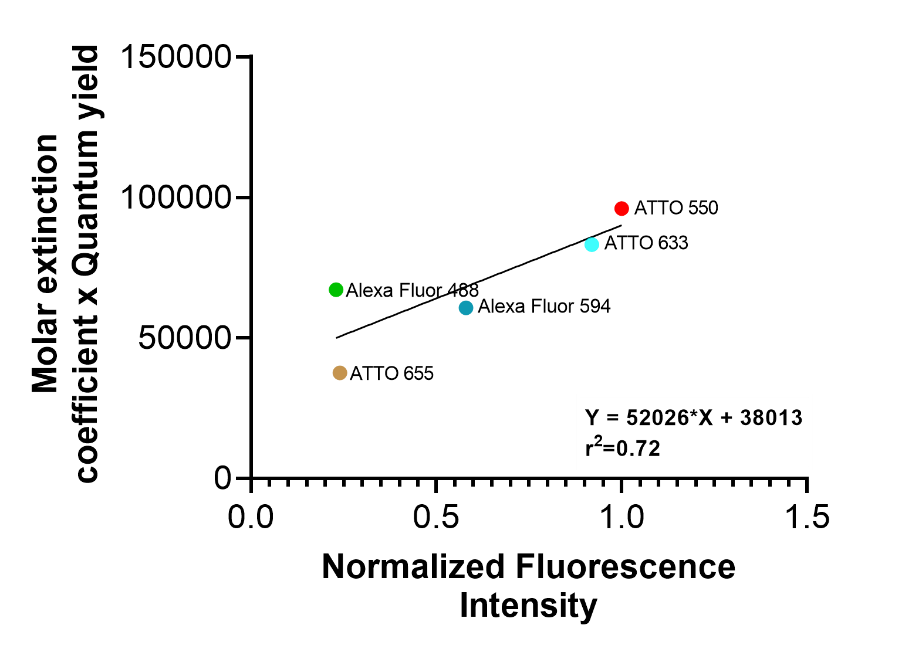


**Figure S1.** Correlation between the normalized fluorescence intensity of each fluorochrome and the product of both its molar extinction coefficient and its quantum yield (relative brightness). The molar extinction coefficients and the quantum yields were obtained from data available by fluorochromes companies. The product of molar extinction coefficient and quantum yield for Alexa Fluor 405 and Alexa Fluor 514 is not represented in figure as its quantum yields are not available on literature.
